# Structural Diversity of Mitochondria in the Neuromuscular System across Development Revealed by 3D Electron Microscopy

**DOI:** 10.1002/advs.202411191

**Published:** 2025-03-06

**Authors:** J. Alexander Bae, Myung‐kyu Choi, Soungyub Ahn, Gwanho Ko, Daniel T. Choe, Hyunsoo Yim, Ken C. Nguyen, Jinseop S. Kim, David H. Hall, Junho Lee

**Affiliations:** ^1^ Research Institute of Basic Sciences Seoul National University Seoul 08826 Republic of Korea; ^2^ Department of Biological Sciences Seoul National University Seoul 08826 Republic of Korea; ^3^ Dominick P. Purpura Department of Neuroscience Albert Einstein College of Medicine Bronx NY 10461 USA; ^4^ Department of Biological Sciences Sungkyunkwan University Suwon 16419 Republic of Korea

**Keywords:** C. elegans, deep learning, development, electron microscopy, fluorescence imaging, mitochondria, nervous system

## Abstract

As an animal matures, its neural circuit undergoes alterations, yet the developmental changes in intracellular organelles to facilitate these changes is less understood. Using 3D electron microscopy and deep learning, the study develops semi‐automated methods for reconstructing mitochondria in *C*. *elegans* and collected mitochondria reconstructions from normal reproductive stages and dauer, enabling comparative study on mitochondria structure within the neuromuscular system. It is found that various mitochondria structural properties in neurons correlate with synaptic connections and these properties are preserved across development in different neural circuits. To test the necessity of these universal mitochondria properties, the study examines the behavior in *drp‐1* mutants with impaired mitochondria fission and discovers that it causes behavioral deficits. Moreover, it is observed that dauer neurons display distinctive mitochondrial features, and mitochondria in dauer muscles exhibit unique reticulum‐like structure. It is proposed that these specialized mitochondria structures may serve as an adaptive mechanism to support stage‐specific behavioral and physiological needs.

## Graphical abstract

Mitochondria across the entire neuromuscular system have been comprehensively reconstructed at different developmental stages using 3D electron microscopy. Fundamental structural principles related to synaptic function are preserved across development, and these morphologies are adapted to ensure effective neural circuit function. Notably, the alternative stage, dauer, exhibits distinct structural properties in both neurons and muscles.

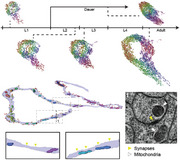

## 1 Introduction

The neural circuit undergoes continuous changes throughout development in various brain regions, adapting for more complex computations and to perform more resilient behavior.^[^ ^1^, ^2^ ^]^ This functional adaptation across development is accompanied by structural alterations, resulting in changes to both neuronal morphology and connectivity between neurons.^[^ ^3^, ^4^, ^5^, ^6^, ^7^, ^8^ ^]^ To induce changes in neuronal morphology and connectivity, numerous intracellular organelles participate in the process, with mitochondria playing a central role through trafficking and reshaping.^[^ ^9^, ^10^, ^11^, ^12^, ^13^ ^]^ However, less is known about how mitochondria structure evolves throughout development to support diverse neural circuits and cellular processes.

3D electron microscopy (EM) provides a distinctive advantage in studying structural properties of mitochondria due to its superior resolution. It allows us to study not only the detailed morphology of individual mitochondrion but also the overall organization of all mitochondria in the volume. Besides, it enables us to relate the mitochondria structural properties with other cellular features such as neurite morphology and synapses since it contains various structural information.

Previously, a number of studies investigated mitochondria structure using 3D EM in mammalian neurons.^[^ ^14^, ^15^, ^16^, ^17^, ^18^, ^19^, ^20^, ^21^ ^]^ However, as mammalian neural circuits are quite large, most studies were limited to a small number of neurons or to selected regions of the brain. In invertebrates, a comprehensive study on mitochondria structure in *Drosophila* has been reported recently; however, this study did not include analysis of mitochondria in muscle cells.^[^ ^22^ ^]^ In order to gain deeper insights into the connection between mitochondria structure and neural circuit function, it is essential to investigate mitochondria structure within an entire system from sensory neurons to muscle cells as there could be regional differences in the neuromuscular system.

Besides, most EM studies on mitochondria are limited to a single time point.^[^ ^14^, ^15^, ^16^, ^18^, ^22^ ^]^ When multiple time points are examined, the studies compare only a few broadly defined intervals like young adult versus aged.^[^ ^17^, ^20^, ^21^ ^]^ However, these time points are rather ill‐defined, making it difficult to identify stereotypic cellular or circuit functions at each interval. In contrast, *C*. *elegans* developmental stages are clearly distinguishable, and numerous studies have reported findings on stage‐specific cellular physiology and behavior in an alternative developmental stage.^[^ ^23^, ^24^, ^25^, ^26^, ^27^, ^28^, ^29^ ^]^

**Figure 1:**
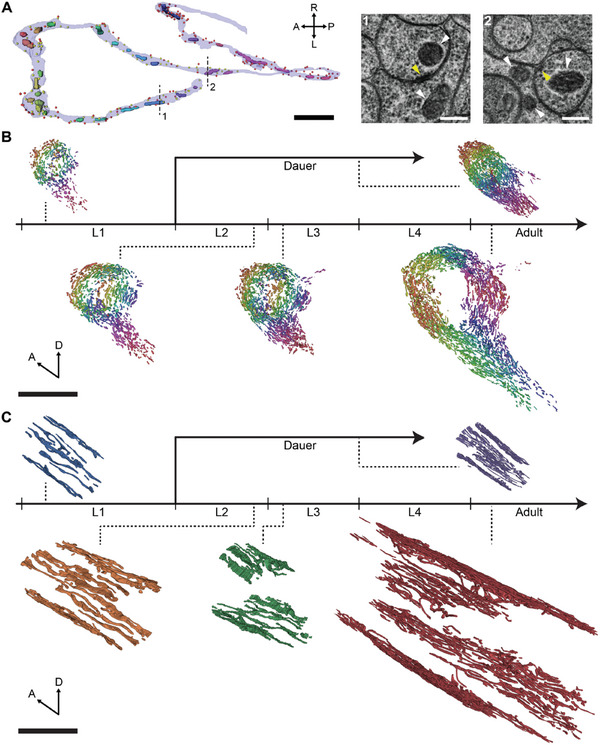
Comprehensive mitochondria reconstructions of *C*. *elegans* across development. A) Mitochondria reconstructions in a sample neuron, RIAR, in the dauer stage with presynaptic sites (i.e., active zones; yellow circles) and postsynaptic sites (red circles) labeled (left). Parts of thin sections at the locations indicated by dashed lines (right) including mitochondria (white arrows) and active zones (yellow arrows). B,C) Dense neuronal mitochondria (B) and body wall muscle mitochondria (C) reconstructions in different developmental stages: L1, L2, L3, adult, and dauer. A) Scale bars: 2 µm (black), 200 nm (white). B,C) Scale bars: 10 µm. A,B) Different mitochondria are denoted by different colors. C) Colors of mitochondria indicate their stage.

Here we use 3D EM and *C*. *elegans* as a model organism to answer the above questions as it becomes feasible to conduct a comprehensive analysis of mitochondria structure within an entire neuromuscular system across development. We have densely reconstructed mitochondria from 3D EM images in the *C*. *elegans* body near the nerve ring for various developmental stages from L1 to adult, and dauer stage (**Figure** **1**).^[^ ^7^, ^30^ ^]^ Our EM reconstructions provide us with opportunities to study detailed mitochondria structure in both neurons and muscle cells across multiple developmental stages from the initial L1 stage after the egg hatch to adult, and also the alternative long‐lived stage, the dauer. As a result, we found that there exist fundamental principles in mitochondria structure that are preserved across development. In addition, we have tested that this specific structure is necessary to operate the neural circuit resulting in the intended behavior. Lastly, we observed that mitochondria in the dauer stage exhibit distinct morphology in both neuronal and non‐neuronal cells, providing insights into their role during the energy‐conserving phase.

## 2 Results

### 2.1 Comprehensive Mitochondria Reconstructions across Development

Mitochondria have distinctive visual features in EM (Figure 1A) so it is possible to visually identify mitochondria in EM. Consequently, we have trained a convolutional neural network (CNN) to automatically detect mitochondria in each image section (Figure S1, Supporting Information; Experimental Section). Then, we reconstructed mitochondria in 3D by combining predictions from adjacent sections (Figure S1, Supporting Information; Experimental Section).

We have densely reconstructed mitochondria in different developmental stages of *C*. *elegans*: L1, L2, L3, adult, and dauer (Figure 1B,C). For normal reproductive stages, we have used publicly available EM image volumes which have been published recently.^[^ ^7^ ^]^ For dauer, we imaged the *C*. *elegans* dauer with length of ∼18 µm using serial‐section EM.^[^ ^30^ ^]^ We have reconstructed all the mitochondria, both in neuronal and non‐neuronal cells like body wall muscles (BWMs), included in the EM volume (Figure 1B,C).

In the neurites of neurons, mitochondria exist in pill‐shaped pieces, and they rarely have branching (Figure 1B). The total number of mitochondria in neurons had a linearly increasing trend from L1 to adulthood, approximately a five‐fold increase (Figure S2A, Supporting Information; L1: *n* = 452, L2: *n* = 763; L3: *n* = 1009, Adult: *n* = 1775). This is similar to the increase in neurite length and body length from L1 to adulthood.^[^ ^7^ ^]^ In the dauer stage, neurons contained about 20% more mitochondria than neurons in L3 (*n* = 1227), which is a similar stage in terms of developmental cycle (Figure S2A, Supporting Information).

The size of mitochondria increased from L1 to L2, then the size of mitochondria saturated to a similar level from L2 to adult (Figure S2B, Supporting Information). The dauer neurons contained substantially smaller mitochondria while the lengths of mitochondria were comparable (Figure S2B,C, Supporting Information). As more and larger mitochondria exist in adult neurons due to their larger volume, mitochondria in the nerve ring showed eightfold increase in total volume (Figure S2D–F, Supporting Information). The dauer stage showed similar value with stage L2 (Figure S2D, Supporting Information).

In the BWMs, mitochondria exist in elongated form spanning over the muscles, which is expected as the mitochondria need to provide energy in every location along the body. As the worm develops from L1 to adult, the mitochondria form more branches leading to more complex shapes with aligned strands, similar to the previous report (Figure 1C).^[^ ^31^, ^32^ ^]^ The BWM mitochondria in dauer has more branching as in adults but are composed of sparser strands which we will elaborate in the later section (Figure 1C).

### 2.2 Mitochondria Structure is Related to Synaptic Connections

**Figure 2:**
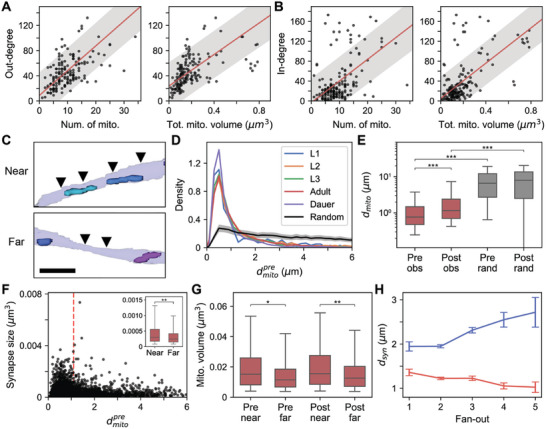
Mitochondria location is related to synaptic connections. A) Out‐degree is positively correlated with number of mitochondria in neuron (left: *n* = 161, *r* = 0.65, *p* = 1.70 × 10^−20^; Pearson correlation) and sum of the mitochondria volume in neuron (right: *n* = 161, *r* = 0.62, *p* = 2.46 × 10^−18^; Pearson correlation). RIAs are not shown for visualization purposes as they have outlying out‐degree (see Figure S3, Supporting Information). B) Same with (A) for in‐degree (left: *n* = 179, *r* = 0.56, *p* = 1.89 × 10^−16^; right: *n* = 179, *r* = 0.68, *P* ≈ 0; Pearson correlation). C) Examples of mitochondria and synapses (arrowheads) positioned proximally (top) and distally (bottom). D) Distance to nearest mitochondria from presynaptic sites in different developmental stages (color lines) and distance to randomly assigned mitochondria (black line). Area under the curve is normalized to be equal to 1. E) Distance to postsynaptic sites is greater than distance to presynaptic sites (n_pre_ = 3677, n_post_ = 7099, *P* ≈ 0) and distance to randomly assigned mitochondria is significantly larger than distance to the nearest mitochondria (*P* ≈ 0). F) Active zones near (below red dashed) mitochondria are larger (inset) than those farther away (n_near_ = 2408, n_far_ = 1269, *p* = 3.96 × 10^−12^). G) Mitochondria near (below red dashed) synapses are larger (inset) than those farther away for both pre‐ (left; n_near_ = 1274, n_far_ = 219, *p* = 6.16 × 10^−5^) and postsynaptic sites (right; n_near_ = 1249, n_far_ = 465, *p* = 1.19 × 10^−6^). H) Mitochondria are located closer to presynaptic sites (red) and farther away from postsynaptic sites (blue) for synapses with larger fan‐out (Pre: n_1_ = 514, n_2_ = 2303, n_3_ = 698, n_4_ = 125, n_5_ = 35; Post: n_1_ = 500, n_2_ = 4383, n_3_ = 1785, n_4_ = 361, n_5_ = 66). A,B) Line: linear fit, shade: 80% prediction interval. C) Scale bar: 1 µm. D) Line: mean, shade: 95% confidence interval. E–G) Center line: median, box: interquartile range, whiskers: 5th and 95th percentile. *P < 10^−4^, **P < 10^−5^, ***P ≈ 0; Wilcoxon rank‐sum test. A,B,E,F,G,H) Adult data are shown as representative.

Mitochondria are known to be closely associated with synaptic connections. Since we are provided with both the morphology and spatial locations of synapses and mitochondria, it is possible to investigate the relation between them. As a result, we found out‐degree, the number of outgoing synapses, is correlated with the number of mitochondria (*n* = 161, *r* = 0.65, *P* < 10^−19^; Pearson correlation) and the total amount of mitochondria (*n* = 161, *r* = 0.62, *p* < 10^−17^; Pearson correlation) in a neuron in all developmental stages (**Figure** **2**A; Figure S3A, Supporting Information). Moreover, the number of mitochondria (*n* = 179, *r* = 0.56, *p* < 10^−15^; Pearson correlation) and the total amount of mitochondria (*n* = 179, *r* = 0.68, *P* ≈ 0; Pearson correlation) are also correlated with in‐degree, the number of incoming synapses. (Figure 2B; Figure S3B, Supporting Information). These results suggest that neurons with more mitochondria tend to form more synaptic connections.

Nevertheless, the correlation between mitochondria morphology and out‐degree does not explain how mitochondria and synapses are spatially correlated, as mitochondria might be concentrated at certain locations in neurites regardless of synapse locations. Therefore, we measured the distance from each active zone ($d_{mito}^{pre}$) and each postsynaptic site ($d_{mito}^{post}$) to the nearest mitochondria. Looking at the distribution of these distances, we were able to observe a peak within 1 µm in every developmental stage (Figure 2C,D). Considering the diameter of a bouton in the neurite is approximately in the range of 1 to 2 µm, this means there are mitochondria located at the same bouton where there are outgoing or incoming synapses. Consistent with previous results in *Drosophila*, we found that distances to postsynaptic sites are significantly larger than distances to active zones (Figure 2E; Figure S3C, Supporting Information).^[^ ^22^, ^33^ ^]^ To test this result did not occur by chance, we have assigned a random mitochondrion within a neuron for every active zone (Experimental Section) and measured the distance to the nearest mitochondria. We were able to see that the distribution of distances in the randomized configurations were relatively more uniform and the distances in our data were significantly smaller in the observed configuration for both pre‐ and postsynaptic sites (Figure 2D,E; n_pre_ = 3677, n_post_ = 7099, *P* ≈ 0; Wilcoxon rank‐sum test).

Mitochondria support synaptic transmission by regulating neurotransmitter release by several mechanisms like calcium buffering. Therefore, mitochondria proximity to synapses could be important and we hypothesized mitochondria would be closely located to support larger synapses (i.e., larger active zones). Indeed, we found that large synapses tend to have the nearest mitochondria closer than smaller synapses (Figure 2F; Figure S4A, Supporting Information). Most of the synapses, over 95%, had similar synapse size, less than 0.001 µm^3^ but there were large synapses which have a diverse range of sizes (Figure 2F). Synapses that have mitochondria nearby (Experimental Section) were significantly larger than synapses with mitochondria farther away (Figure 2F; n_near_ = 2408, n_far_ = 1269, *P* < 10^−11^; Wilcoxon rank‐sum test). This result is consistent with study in mammalian neurons,^[^ ^15^ ^]^ providing additional evidence for the relationship between mitochondria proximity and synapse efficacy. Vice versa, we measured the distance to pre‐ ($d_{syn}^{pre}$) and postsynaptic ($d_{syn}^{post}$) from the nearest mitochondria and observed mitochondria located closer to synapses were larger, indicating larger mitochondria are needed to operate larger synapses (Figure 2G, Figure S4B,C, Supporting Information; pre: n_near_ = 1274, n_far_ = 219, *P* < 10^−4^, post: n_near_ = 1249, n_far_ = 465, *P* < 10^−5^; Wilcoxon rank‐sum test).

*C*. *elegans* is a polyadic animal, meaning a single active zone can send neurotransmitters to multiple partners. From the above results, we hypothesized that active zones with higher fan‐out, number of postsynaptic partners per active zone, would have larger mitochondria nearby as active zones with larger fanouts have larger synapses (Figure S5A, Supporting Information). As expected, the nearest mitochondria were larger and closer to the synapses as fan‐out increased (Figure 2H; Figure S5B,C, Supporting Information). On the contrary, the nearest mitochondria were farther away at the postsynaptic sites when they shared input with more cells (Figure 2H; Figure S5B, Supporting Information). All the relations were consistent among different developmental stages (Figures S3–S5, Supporting Information).

### 2.3 Axonal Mitochondria are Shorter than Dendritic Mitochondria

**Figure 3:**
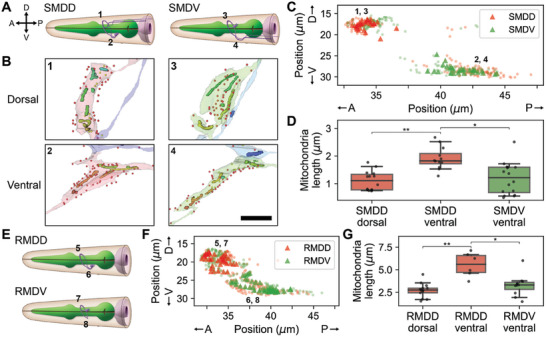
Axonal mitochondria are shorter. A) Diagram of SMDD (left) and SMDV (right) neurons. B) Reconstructed mitochondria in adult SMDD (left) and SMDV dorsal (top) and ventral (bottom) boutons. Number indicates locations marked in (A). Presynaptic (yellow) and postsynaptic (red) sites are marked with dots. C) Distribution of outgoing (triangle) and incoming (circle) synapses of SMDD (red) and SMDV (green). Numbers are locations of numbered boxes in (B). D) Mitochondria are longer in the ventral bouton of SMDD, where it only receives inputs (left, n_d_ = 14, n_v_ = 12, *p* = 0.00011). Within the ventral boutons, mitochondria are longer in SMDD (right, n_SMDD_ = 12, n_SMDV_ = 16, *p* = 0.00116). E) Diagram of RMDD (top) and RMDV (bottom) neurons. F) Same with (C) for RMDD (red) and RMDV (green). G) Mitochondria are longer in the ventral bouton of RMDD, where it only receives inputs (left, *p* = 0.00086). Within the ventral boutons, mitochondria are longer in RMDD (right, *p* = 0.00356). A,E) Diagrams are reproduced with permission.^[^ ^34^ ^]^ A: anterior, P: posterior, D: dorsal, V: ventral. B) Scale bar: 2 µm. D,G) Center line: median, box: interquartile range, whiskers: 5th and 95th percentile. *P < 0.01, **P < 0.001; Wilcoxon rank‐sum test.

Mitochondria morphologies differ in different compartments (e.g., axon, dendrite, and soma) in mammalian neurons^[^ ^18^, ^35^, ^36^ ^]^ as each compartment serves a different function. In *C*. *elegans* neurons, roles of different compartments are merged as the same neurite can both send and receive signals. However, there are exceptions in symmetric motor neurons. For example, it is known that SMDD and SMDV have outgoing synapses only at boutons in dorsal and ventral regions of the arbor (**Figure** **3**A–C; Figure S6A–D, Supporting Information) respectively, which leads to compartmentalized activity in RIA.^[^ ^37^ ^]^ Unlike outgoing synapses, the incoming synapses exist at both boutons (Figure 3A–C). Other classes of motor neurons such as RMD and SMBD neurons also show compartmentalized synapse distribution (Figure 3E,F; Figure S7A–D, Supporting Information). A similar pattern is observed in RIA neurons as well (Figure S7E,F, Supporting Information).

Due to compartmentalized synapse distribution, it is feasible to isolate the neurite region with only incoming synapses, like dendrites, and study whether there is any difference in mitochondria structures. Here, we will define mitochondria in this region with only incoming synapses as “dendritic” and the ones in the other regions as “axonal” for convenience even though the boutons have mixed pre‐ and postsynaptic sites. We discovered that dendritic mitochondria are longer compared to axonal mitochondria in SMD neurons (Figure 3B; Figure S6, Supporting Information). We have verified this by comparing mitochondria in SMDD dorsal (axonal) and SMDD ventral (dendritic) boutons (Figure 3D; n_d_ = 14, n_v_ = 12, *p* = 0.00011; Wilcoxon rank‐sum test). As this result could be due to the difference in the bouton size, when we compared the ventral boutons of SMDD and SMDV, the mitochondria in SMDV ventral (axonal) boutons were smaller than mitochondria in SMDD ventral (dendritic) boutons (Figure 3D; n_SMDD_ = 12, n_SMDV_ = 16, *p* = 0.00116; Wilcoxon rank‐sum test). Similar results have been tested with RMD neurons and found mitochondria in RMDD ventral boutons are longer than those in RMDD dorsal (n_d_ = 13, n_v_ = 6, *p* = 0.00086; Wilcoxon rank‐sum test) and RMDV ventral boutons (Figure 3G; n_SMDD_ = 6, n_SMDV_ = 11, *p* = 0.00356; Wilcoxon rank‐sum test). This result is consistent with previous findings that dendritic mitochondria tend to be larger and longer.^[^ ^18^, ^35^, ^36^ ^]^ This structural property is a fundamental principle that can be found in all developmental stages (Figure S6, Supporting Information).

### 2.4 Mitochondria Morphology is Adapted to Accommodate Neural Circuit Function

Above, we have seen that axonal mitochondria have shorter morphology, and mitochondria are spatially related to synapses. Therefore, we questioned whether specific mitochondria morphology and localization is necessary for proper synaptic transmission, thereby important for intended behavioral output. SMD neurons have been known to be involved in omega turns or sharp directional changes as they activate head and neck muscles.^[^ ^38^, ^39^ ^]^ Assuming mitochondria morphology is adapted to support synaptic transmission, we hypothesized disruption of mitochondria structures in SMD neurons would interrupt innervation of body wall muscles, inhibiting the turning behavior.

**Figure 4:**
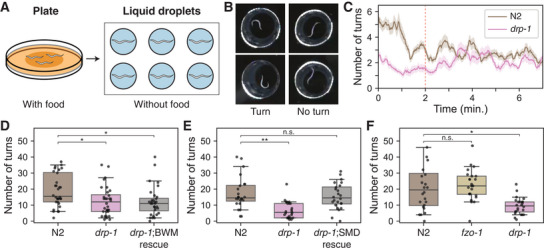
Mitochondria morphology is adapted to accommodate neural circuit function. A) Behavior experiment setup. Worms were transferred from the plate with food to the liquid droplets without food, then the number of turns were counted. B) Example images of worm's behavior in the liquid droplet (left: turning, right: not turning). C) Number of turns for N2 wild‐type (brown) and *drp‐1* mutants (pink) recorded over time (red dashed: 2‐minute mark). D) Total number of turns in the first 2 minutes for N2 wild‐type, *drp‐1* mutants, and BWM‐specific rescue model (*n* = 32, *P_drp‐1_* = 0.009, *P*_rescue_ = 0.003). *drp‐1* mutants exhibit significantly lower number of turns compared to the wild‐type and impaired behavior in *drp‐1* mutants are not recovered in the BWM‐specific rescue model. E) Same with (D) for SMD neurons and the behavior is recovered in the SMD‐specific rescue model (*n* = 24, *P_drp‐1_* = 1.64 × 10^−5^, *P*_rescue_ = 0.66). F) Same with (D) for *fzo‐1* mutants (*n* = 24, *P_fzo‐1_* = 0.49, *P_drp‐1_* = 0.003). *fzo‐1* mutants do not show impaired turning rate as *drp‐1* mutants. D–F) Center line: median, box: interquartile range, whiskers: 5th and 95th percentile. *P < 0.01, **P < 0.0001; Wilcoxon rank‐sum test.

In this study, we studied a kind of exploratory behavior, local search, to investigate how mitochondria structure affects the behavior (**Figure** **4**A,B).^[^ ^39^ ^]^ When the animals are removed from food, they make sharp turns to search for food, mediated by SMD neurons, similar to biased random walk.^[^ ^39^, ^40^ ^]^ As it has been previously reported, animals showed a high turning rate due to local search behavior immediately after they have been transferred to an environment without food (Figure 4C). Then the turning rate relaxed to the base rate where they stopped showing local search behavior (Figure 4C). According to our hypothesis, we expected this local search behavior would be impaired when the mitochondria structure is disrupted.

To test this idea, we checked the local search behavior in the mutants important for the mitochondria morphology *drp‐1*, a mitochondria fission factor. In fact, *drp‐1* mutants, where mitochondria fission is interrupted, showed a defect in increased turning rate during local search behavior (Figure 4C). This result suggests that mitochondria morphology and localization regulated by the mitochondria fission factor is important for proper synaptic function in SMD neurons.

To quantitatively compare the local search behavior, we counted the total number of turns within the first 2 minutes after the worms were transferred, where an increased turning rate was observed in wild‐type animals (Figure 4C). The total number of turns were significantly lower in *drp‐1* mutants compared to the wild‐type as expected (Figure 4D–F; n_N2_ = 32, 24, 24, n_drp1_ = 32, 24, 24, *p* = 0.009, *p* = 1.64 × 10^−5^, *p* = 0.003; Wilcoxon rank‐sum test). However, *drp‐1* mutants have broken mitochondria structure in various cells. We first tested whether this deficit in behavior is caused by the disruption of mitochondria morphology in muscle cells as they are the cells that execute the behavior. However, the total number of turns in BWM‐specific rescue of *drp‐1* mutants were not significantly different from that of *drp‐1* mutants (Figure 4D; n_N2_ = 32, n_rescue_ = 32, *p* = 0.003; Wilcoxon rank‐sum test).

With BWMs excluded from the candidates, we then moved onto our original hypothesis, that this behavior deficit is due to the dysfunction in SMD neurons, which fails to innervate BWMs. We created SMD‐specific rescue of *drp‐1* mutants and were able to see the local search behavior recovered to the normal level (Figure 4E; n_N2_ = 24, n_rescue_ = 24, *p* = 0.66).

Mitochondria undergo dynamic structural and functional regulation through mitochondria quality control mechanism, involving both mitochondria fission and fusion.^[^ ^41^ ^]^ Unlike mitochondria fission factor (*drp‐1*) mutants, the mitochondria fusion factor (*fzo‐1*) mutants showed no impairment in turning behavior (Figure 4F). While mitochondrial function is compromised in both *drp‐1* and *fzo‐1* mutants, they lead to different morphological consequences, and behavioral deficits were observed only in fission mutants. These results imply mitochondria structure is adapted to accommodate proper synaptic transmission in neurons to maintain effective functioning of the neural circuit.

### 2.5 Dauer Inter‐ and Motor Neurons Show Distinctive Mitochondrial Features

Beyond the fundamental structural properties of mitochondria conserved across development, are there any differences? Since we have densely reconstructed mitochondria in diverse developmental stages, it enables us to conduct stage‐wise quantitative comparison of mitochondria structure. As mitochondria structure is related to synaptic connections (Figure 2), investigating mitochondria morphologies of neurons in different stages could provide insights in understanding the differences in neural circuits.

**Figure 5:**
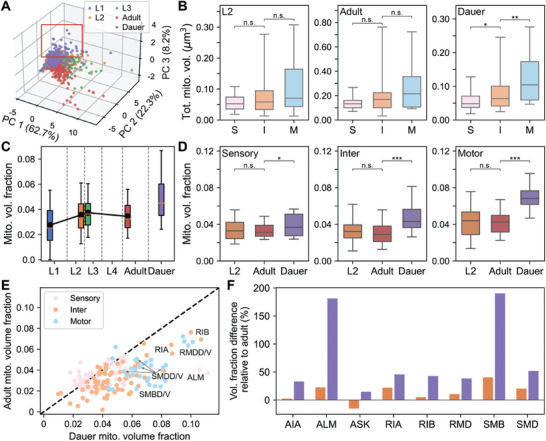
Dauer inter‐ and motor neurons show distinctive mitochondrial features. A) Principal component embeddings of neuronal mitochondrial features show group of dauer neurons clustered outside (red box). B) Distribution of total mitochondria volume in neurons per type for L2 (left; *p* = 0.35, *p* = 0.15), adult (middle; *p* = 0.12, *p* = 0.09), and dauer (right; *p* = 0.014, *p* = 0.004). C) Distribution of mitochondria volume fraction across development. D) Distribution of mitochondria volume fraction in L2, adult, and dauer for sensory (left; *p* = 0.772, *p* = 0.045), inter‐ (middle; *p* = 0.333, *p* = 2.99 × 10^−8^), and motor (right; *p* = 0.778, *p* = 3.69 × 10^−8^) neurons. E) Comparison between mitochondria volume fraction of dauer and adult. Dauer shows higher mitochondria volume fraction relative to other developmental stages. Dashed line indicates y = x line. F) Difference in mitochondria volume fraction compared to selected neurons in adult for L2 (orange) and dauer (purple). Volume fraction difference is larger in the dauer stage. C) Black line: mean. B–D) Center line: median, box: interquartile range, whiskers: 5th and 95th percentile. B,D) n_sen_ = 61, n_int_ = 69, n_mot_ = 32, *p < 0.05, **p < 0.01, ***p < 10^−7^; Wilcoxon rank‐sum test.

We have computed various mitochondrial features including total mitochondria volume, surface area, volume fraction, length, and mitochondrial complexity index (MCI).^[^ ^42^ ^]^ We initially explored an overview of mitochondrial features by projecting all mitochondrial features onto principal components. As a result, we observed that several dauer neurons are separately clustered from the majority, indicating that neuronal mitochondria in dauer are distinctive (**Figure** **5**A; Figure S8A, Supporting Information).

Neurons in *C*. *elegans* are classified into three types – sensory, inter‐, and motor neurons – depending on their functions. Variability in function can be encoded in their arbors as the cell shapes of different types differ and mitochondria structure can also differ accordingly. In most of the stages, no significant difference in the distribution of total mitochondria volume was observed between different cell types, even though there were some interneurons and motor neurons that possess large amounts of mitochondria (Figure 5B; Figure S8B, Supporting Information). On the other hand, motor neurons in dauer had greater total mitochondria volume on average compared to interneurons and sensory neurons (Figure 5B; n_sen_ = 61, n_int_ = 69, *n*_mot_ = 32, *p* = 0.004; Wilcoxon rank‐sum test). Moreover, interneurons in dauer had significantly more mitochondria than sensory neurons (Figure 5B; *p* = 0.014; Wilcoxon rank‐sum test).

Still, we lack evidence to draw conclusions regarding stage‐wise comparisons because the size of neurons differs between stages, and it is obvious that larger cells are likely to have more mitochondria (Figure S2D–F, Supporting Information). In order to compensate for this caveat, we introduced a measure, mitochondria volume fraction, where we divide the total mitochondria volume in a neuron by the neuron arbor volume. Unlike the mitochondria volume, mitochondria volume fraction was constant in normal reproductive stages, from L1 to adult stages except for the dauer stage, where the mitochondria volume fraction was exceptionally high (Figure 5C). When we analyze per cell types, the mitochondria volume fraction of neurons in dauer were significantly greater in interneurons (n_int_ = 69, *p* = 0.333, *p* = 2.99 × 10^−8^; Wilcoxon rank‐sum test) and motor neurons (*n*_mot_ = 32, *p* = 0.778, *p* = 3.69 × 10^−8^) while the difference was negligible in sensory neurons (Figure 5D; n_sen_ = 61, *p* = 0.772, *p* = 0.045; Wilcoxon rank‐sum test). As the amount of mitochondria is correlated with number of connections, this result could imply increased role of motor neurons in neural circuits of dauer compared to normal reproductive stages, which is consistent with recent findings in *C*. *elegans* dauer connectome.^[^ ^30^ ^]^

To verify that this difference of mitochondria volume fraction is true for the same neuron in different stages, we looked at the mitochondria volume fraction of individual neurons (Figure 5E). Mitochondria volume fractions of neurons in dauer were substantially larger for many neurons (Figure 5E,F) while the volume fractions of neurons in other stages were similar (Figure S8D,E, Supporting Information). As we saw from the distributions, many interneurons and motor neurons tend to have higher mitochondria volume fraction, especially neurons involved in head and neck movement (Figure 5E,F).^[^ ^39^ ^]^

### 2.6 Dauer Cholinergic and Glutamatergic Neurons Show Distinctive Mitochondrial Features

**Figure 6:**
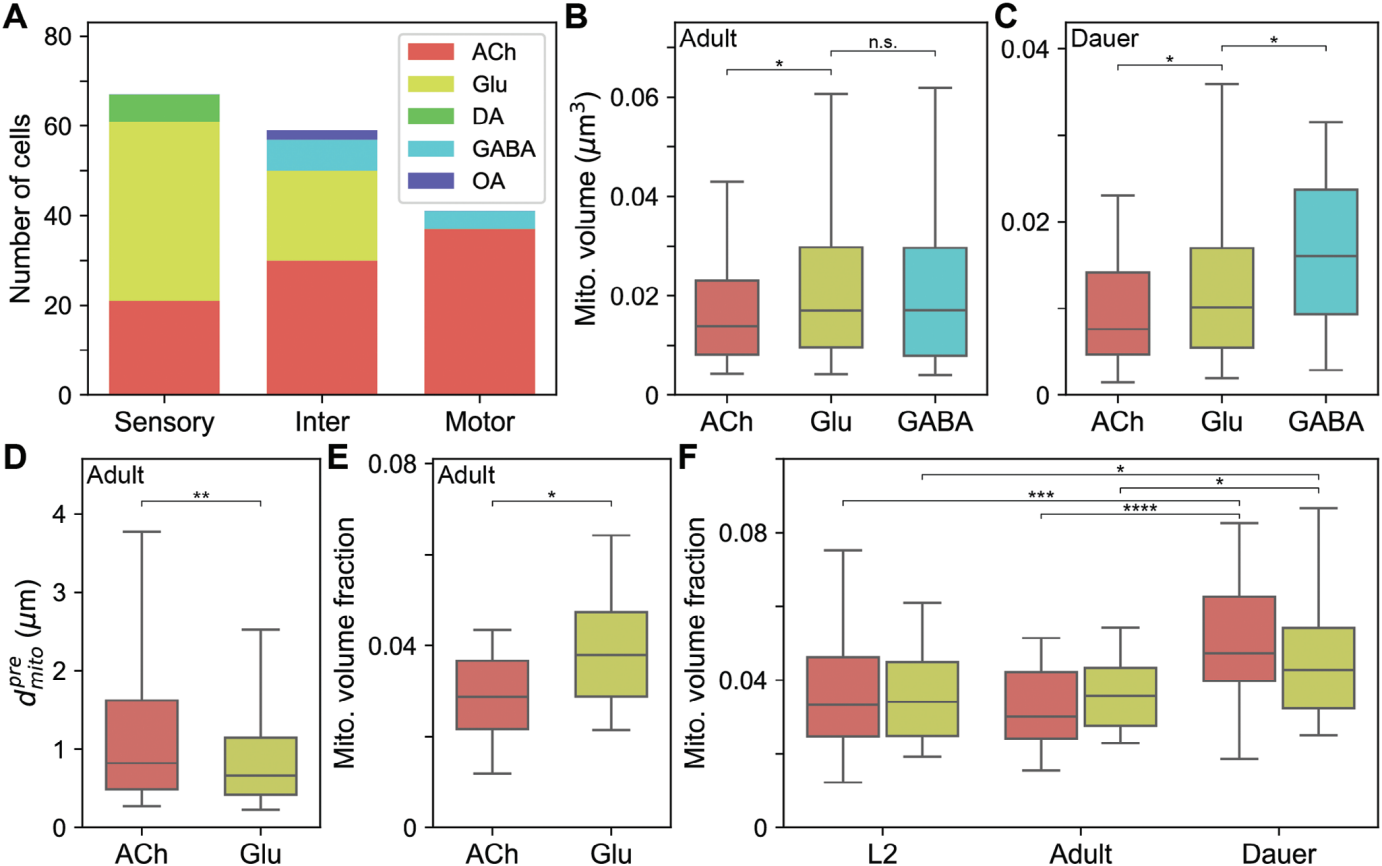
Dauer cholinergic and glutamatergic neurons show distinctive mitochondrial features. A) Proportion of neuron types by neurotransmitter classification for each cell type. B) Glutamatergic interneurons have larger mitochondria than cholinergic interneurons (n_ACh_ = 267, n_Glu_ = 308, n_GABA_ = 108, *p* = 0.001, *p* = 0.797). C) Same with (B) but GABAergic interneurons have larger mitochondria than glutamatergic interneurons in dauer (n_ACh_ = 186, n_Glu_ = 199, n_GABA_ = 48, *p* = 0.002, *p* = 0.007). D) Glutamatergic interneurons have mitochondria closer to active zones than cholinergic interneurons (n_ACh_ = 428, n_Glu_ = 845, *p* = 4.45 × 10^−6^). E) Glutamatergic interneurons have higher mitochondria volume fraction than cholinergic interneurons (n_ACh_ = 30, n_Glu_ = 20, *p* = 0.007). F) Dauer cholinergic (*n* = 78, 85, 85, *p* = 7.95 × 10^−7^, *p* = 3.48 × 10^−9^) and glutamatergic neurons (*n* = 58, *p* = 0.003, *p* = 0.003) have higher mitochondria volume fraction. A–E) Ach: Acetylcholine, Glu: Glutamate, DA: Dopamine, OA: Octopamine. B–F) Center line: median, box: interquartile range, whiskers: 5th and 95th percentile. *p < 0.01, **p < 10^−5^, ***p < 10^−6^, ****p < 10^−8^; Wilcoxon rank‐sum test.

Given the critical role of mitochondrial function in neurotransmitter release, it is reasonable to assume that mitochondria structure may vary depending on neurotransmitter identities. Besides neuronal types, neurons can be classified based on the neurotransmitters they release, with the possibility of diverse neurotransmitters within the same type.^[^ ^38^ ^]^ In *C*. *elegans*, most neurons release acetylcholine (ACh) and glutamate (Glu), with sensory and interneurons primarily consisting of cholinergic and glutamatergic types (**Figure** **6**A). Additionally, a few sensory neurons release dopamine, while several interneurons release GABA (Figure 6A). For this study, we restricted our analysis on the three predominant neurotransmitters – ACh, Glu, and GABA – since neurons releasing other neurotransmitters are too few to draw reliable conclusions.

We focused our analysis on interneurons because these neurons primarily connect with other neurons rather than non‐neuronal cells. Among interneurons, we found that mitochondria in glutamatergic and GABAergic neurons have larger mitochondria than those in cholinergic neurons in normal reproductive stages (Figure 6B; Figure S9A, Supporting Information; n_ACh_ = 267, n_Glu_ = 308, n_GABA_ = 108, *p* = 0.001, *p* = 0.797; Wilcoxon rank‐sum test). Alternatively, GABAergic interneurons possess even larger mitochondria than glutamatergic interneurons in the dauer stage (Figure 6C; n_ACh_ = 186, n_Glu_ = 199, n_GABA_ = 48, *p* = 0.002, *p* = 0.007; Wilcoxon rank‐sum test).

Since glutamatergic neurons have larger mitochondria on average, we hypothesized that mitochondria in these neurons might be located closer to synapses. We found that glutamatergic interneurons have the nearest mitochondria more closely located to the active zones (Figure 6D; n_ACh_ = 428, n_Glu_ = 845, *p* = 4.45 × 10^−6^; Wilcoxon rank‐sum test) and have higher mitochondria volume fraction (Figure 6E; n_ACh_ = 30, n_Glu_ = 20, *p* = 0.007; Wilcoxon rank‐sum test) compared to cholinergic interneurons. These findings were also observed in other developmental stages (Figure S9B,C, Supporting Information) and align with previous reports in *Drosophila* whole brain, suggesting this may be a shared characteristic among invertebrates.^[^ ^22^ ^]^

From the above results, it can be inferred that more mitochondria are needed closer to synapses in glutamatergic and GABAergic neurons (Figure 6B–E). Considering GABA is inhibitory neurotransmitter, and glutamate can act as inhibitory neurotransmitter in some neurons like AIB and RIM, this result could be indicating inhibitory interneurons require more energy per cell compared to other excitatory neurons.^[^ ^22^, ^43^ ^]^

Although structural properties of mitochondria are generally consistent across development among neurons releasing the same neurotransmitter, variations were also noted between different stages. We noticed that both cholinergic (n_L2_ = 78, n_adult_ = 85, n_dauer_ = 85, *p* = 7.95 × 10^−7^, *p* = 3.48 × 10^−9^; Wilcoxon rank‐sum test) and glutamatergic (*n* = 58, *p* = 0.003, *p* = 0.003; Wilcoxon rank‐sum test) neurons in dauer, regardless of the cell types, show higher mitochondria volume fraction than normal reproductive stages (Figure 6F). Acetylcholine and glutamate are known to play crucial roles during the dauer stage such as modulating behavioral responses specific to the dauer stage. The mitochondria structure could have been adapted to accommodate the stage‐specific needs in cholinergic and glutamatergic neurons.

### 2.7 Dauer Body Wall Muscle Mitochondria Exhibit Distinctive Structure

We have not only reconstructed neuronal mitochondria but also reconstructed those in body wall muscles (BWMs), allowing us to make structural comparisons of BWM mitochondria across development. The BWMs included in the data are head and neck muscles as our study has been centered around the vicinity of the nerve ring.

**Figure 7:**
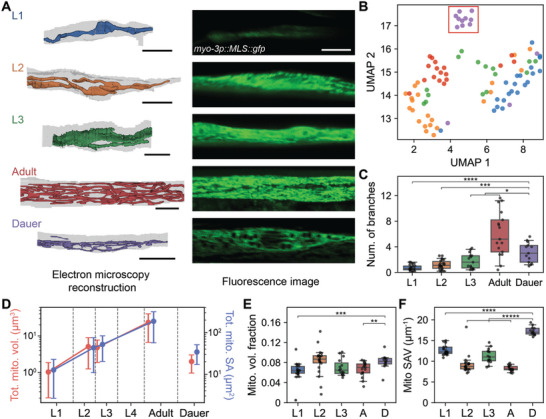
Dauer body wall muscle mitochondria exhibit distinctive structure. A) Reconstructed mitochondria in example body wall muscles (BWMs) in different developmental stages (left) and corresponding fluorescence images of mitochondria in BWM (right). B) UMAP embeddings of BWM mitochondria features show group of dauer cells clustered (red box). C) Number of branches in mitochondria across development (*P*_L1_ = 1.54 × 10^−5^, *P*_L2_ = 0.004, *P*_L3_ = 0.017, *P*_adult_ = 0.03). D) Total mitochondria volume (red) and total mitochondria surface area (blue) across development (mean±SD). E) Mitochondria volume fraction across development (*P*_L1_ = 4.22 × 10^−4^, *P*_L2_ = 0.34, *P*_L3_ = 0.05, *P*_adult_ = 0.003). F) Mitochondria surface area per volume (SAV) across development (*P*_L1_ = 1.21 × 10^−5^, *P*_L2_ = 6.59 × 10^−6^, *P*_L3_ = 7.08 × 10^−6^, *P*_adult_ = 3.75 × 10^−6^). A) Scale bars: 5 µm (black), 5 µm (white). B–F) n_L1_ = 21, n_L2_ = 22, n_L3_ = 15, n_adult_ = 17, n_dauer_ = 13. C,E,F) Center line: median, box: interquartile range, whiskers: 5th and 95th percentile. *P < 0.05, **P < 0.01, ***P < 0.001, ****P < 10^−4^, *****P < 10^−5^; Wilcoxon rank‐sum test.

Interestingly, the morphologies of mitochondria in head and neck muscles undergo substantial changes throughout the development (**Figure** **7**A). In L1, muscle cells contain a solitary strand of mitochondria, and as the worm matures, it branches into additional mitochondria strands (Figure 7A). Once the worm becomes an adult, the mitochondria form a reticulum‐like structure with many strands intermingled (Figure 7A). Similar developmental changes have been observed using fluorescence imaging in BWMs.^[^ ^31^, ^32^ ^]^ In dauer, the BWMs exhibit a distinctive reticulum‐like structure, which distinguishes from the adult stage, where the strands are slimmer and adjacent portions of the muscle belly contain greater empty spaces. (Figure 7A).^[^ ^44^ ^]^ To confirm that our findings are not limited to specific animals, we acquired fluorescence images of mitochondria in head and neck muscles. We have verified that our observation is valid in different animals, further highlighting the distinctive reticulum‐like structure in the dauer stage (Figure 7A).

Since we have high‐resolution reconstructions, we can also quantify the BWM mitochondria morphologies and quantitatively confirm the above results from qualitative investigation. When analyzing various mitochondrial features using UMAP embeddings,^[^ ^45^ ^]^ we found dauer muscle cells form a separate cluster, confirming that mitochondria in the dauer stage display distinctive characteristics (Figure 7B, Experimental Section).

Upon closer quantitative examination, we noted an increased branching (Figure 7B) and an exponential increase in mitochondria volume and surface area from L1 stage to adulthood in BWMs (Figure 7C). Dauer BWMs had larger number of branching with less total mitochondria volume compared to L2 and L3 likely due to enlarged sarcomeres and intracellular structures, although mitochondria surface area remained similar (Figure 7B,C; Figure S10A, Supporting Information).^[^ ^28^, ^44^ ^]^ The MCI also increased from L1 to adulthood, reflecting more complex, network‐like mitochondria structure in adults (Figure 7A; Figure S10B, Supporting Information). Notably, dauer BWM mitochondria exhibited a high MCI due to its reticulum‐like morphology (Figure S10B, Supporting Information).

Given the reduced muscle belly in dauer BWMs, we hypothesized that the proportion of area occupied by mitochondria might be larger. However, the mitochondria volume fraction was not significantly different from other stages, with the exception of L1 (Figure 7E). Instead, the surface area per volume of mitochondria in dauer BWMs was significantly higher than in other stages (Figure 7D,F; n_L1_ = 21, n_L2_ = 22, n_L3_ = 15, n_adult_ = 17, n_dauer_ = 13, *P*_L1_ = 1.21 × 10^−5^, *P*_L2_ = 6.59 × 10^−6^, *P*_L3_ = 7.08 × 10^−6^, *P*_adult_ = 3.75 × 10^−6^; Wilcoxon rank‐sum test). This result reflects the qualitative observation that dauer BWM mitochondria are composed of networks of thin strands (Figure 7A).

Next, we checked whether this dauer‐specific mitochondria structure is preserved even after the dauer exit. Captured with fluorescence imaging, BWM mitochondria in postdauer L4 or postdauer adult stages did not show distinctive reticulum‐like structure as seen in dauer (Figure S10C, Supporting Information). Mitochondria in postdauer L4 and postdauer adult did not have empty spaces in between the strands like dauer (Figure S10A, Supporting Information).

## 3 Discussion

In this study, we present comprehensive dense reconstructions of mitochondria in normal reproductive stages and the dauer stage of *C*. *elegans* using 3D electron microscopy. Utilizing these reconstructed datasets, we conducted a comparative analysis of mitochondria structures throughout development, spanning from birth to adulthood, in both neurons and body wall muscles. Consequently, we were able to propose fundamental mitochondria structural principles shared across different stages as well as stage‐specific mitochondrial features. EM offers a distinct advantage due to its superior resolution, enabling detailed quantitative morphological comparisons that are often not feasible with fluorescence imaging, especially in small animal models such as *C*. *elegans*. Furthermore, our investigation encompassed the examination of mitochondria across the nervous system from sensory neurons to muscles. This provides insights into how mitochondria structure might impact neural circuit function leading to behavioral output. Lastly, we observed unique mitochondria structures in the alternative dauer stage, which nematodes enter under adverse environmental conditions, suggesting potential adaptation of intracellular organelles.

### 3.1 Preserved Fundamental Structural Principles across Development

We observed many structural properties of mitochondria preserved throughout the development, including during the alternative dauer stage. The relationship between the quantity and proximity of mitochondria to the synaptic connectivity demonstrates consistent rules across all stages (Figure 2; Figures S3–S5, Supporting Information). Similarly, compartment‐specific mitochondria morphology is conserved across developmental stages (Figure 3; Figures S6 and S7, Supporting Information). In addition, mitochondria exhibit comparable characteristics in each cell type during normal reproductive stages (Figure 5; Figure S8, Supporting Information), even when cells are classified by the neurotransmitters they release (Figure 6; Figure S9, Supporting Information). Cells and neural circuits have essential functions necessary for basic operational capabilities. Therefore, mitochondria need to follow established principles to support these essential functions.

These principles have been similarly identified in mammalian neurons. Previous studies have found the spatial distribution of synapses and mitochondria along the neurite covary, meaning synapses have mitochondria nearby potentially as an energy source and calcium buffer.^[^ ^15^, ^18^ ^]^ Other studies have found that mitochondria in different compartments have different morphological characteristics. It has been discovered that dendritic mitochondria tend to be longer and larger than axonal mitochondria.^[^ ^18^, ^20^, ^35^, ^36^ ^]^ We now report similar findings in *C*. *elegans* indicating the same structural principle applies even in invertebrates, further supported by a recent comprehensive study of mitochondria in *Drosophila*.^[^ ^22^ ^]^

### 3.2 Mitochondria Morphology and Neural Circuit Function

Our experiments using mitochondria fission and fusion factor mutants confirm that specific mitochondria morphology is important for proper neural circuit function, leading to intended behavior. The turning behavior deficits observed in *drp‐1* mutants, along with the recovered behavior in SMD‐specific rescue models, indicate that mitochondria fission, resulting in shorter mitochondria, is necessary for effective synaptic transmission. However, whether short mitochondria morphology itself is crucial functionally near the synapses remains unclear. Based on prior studies, there exists a possibility that shorter mitochondria may facilitate proper axonal mitochondrial transport to provide sufficient amount of mitochondria near synapses.^[^ ^10^ ^]^ Since the resolution of fluorescence imaging is insufficient to distinguish mitochondria within neurites of the nerve ring, further EM studies in fission and fusion mutants would offer deeper insights into the role of mitochondria morphology in synaptic function.

### 3.3 Increased Mitochondria in the Dauer Neurons for Dauer‐Specific Behavior

The notable increase in mitochondria volume fraction in dauer seems ironic since the worms in this stage display long periods of immotility, but with occasional bursts of fast actions.^[^ ^23^, ^46^ ^]^ It is possible that dauer neurons contain more mitochondria relative to cell size as a preparatory mechanism when the occasion arises. The neurons that stand out with higher mitochondria volume fraction in dauer are interneurons (e.g., RIA, RIB) and motor neurons (e.g., RMD, SMD, SMB) that are known to be involved in the head and neck neuromuscular system (Figure 5E,F). These cells are likely to be the drivers of forward and backward body movements. For instance, SMB neurons determine the amplitude of sinusoidal motion^[^ ^39^ ^]^ and RMD neurons are known to control head‐withdrawal reflex and spontaneous foraging behavior.^[^ ^47^ ^]^ A higher mitochondria density in these cells could enable neurons to rapidly activate synaptic connections, leading to quicker responses to the stimuli in the dauer stage.

Based on the previous observations, the dauer‐specific behavior, nictation, is initiated from the head movement.^[^ ^23^, ^29^ ^]^ The sensory neuron which regulates nictation, IL2, has been found,^[^ ^29^ ^]^ but the rest of the neurons in the neural circuit that generate nictation behavior are still unknown despite recent efforts in dauer connectome.^[^ ^30^ ^]^ Neurons like RIA, RIB, and SMD, which exhibit distinctive mitochondrial features, could be plausible candidates responsible for nictation (Figure 5E,F).

RMD, SMD, and IL2 neurons are all cholinergic, and given acetylcholine's crucial role in the neuromuscular function, it is expected to play an important role during the dauer stage. Glutamatergic neurons, including RIB, are known to modulate behaviors in dauer.^[^ ^48^ ^]^ The finding that both cholinergic and glutamatergic neurons exhibit a high mitochondria volume fraction could be reflecting their important roles in the dauer stage (Figure 6F). Therefore, the neurons with higher mitochondria volume fraction in dauer could be candidates that regulate dauer‐specific behavior, which can be tested using optogenetics in future experiments.^[^ ^30^ ^]^

Synaptic transmission not only influences animal behavior but also affects developmental decision making. Previous study reported that pheromone activates ASK and ADL neurons, which in turn stimulates AIA neurons via glutamatergic transmission to induce dauer entry.^[^ ^49^ ^]^ ASK neurons have high mitochondria volume fraction in dauer as other glutamatergic neurons (Figure 6F). Also, AIA neurons are interneurons that show high mitochondria volume fraction in dauer (Figure 6F), indicating the potential adaptation of mitochondria structure in the dauer stage.

### 3.4 Mitochondria and Lipid Droplets in the Dauer Muscle Cells

Previous studies using 2D cross‐section analysis have shown that dauer muscle mitochondria are present in a compact conformation concentrated in the muscle belly.^[^ ^40^ ^]^ It is presumed that this structural property is necessary to provide large amounts of energy on a short‐term basis to support rapid response behavior.^[^ ^21^ ^]^ This study has not only confirmed this discovery but also extended it by uncovering the 3D structural morphology, including the sparse network‐like structure with thin strands (Figure 7).

Then the question naturally arises, what is present within the gaps amidst the strands? It has been reported that dauer muscle cells contain lipids unlike other developmental stages.^[^ ^40^ ^]^ Indeed, the empty spaces between the mitochondria strands were occupied by relatively large organelles with a phospholipid monolayer, presumed to be lipid droplets, forming intimate contacts with mitochondria (Figure S10A, Supporting Information).

During the dauer stage, *C*. *elegans* shifts from glycolysis to using the glyoxylate cycle within mitochondria for energy production, enabling the conversion of stored fats into glucose.^[^ ^50^ ^]^ The unique reticulum‐like structure of BWM mitochondria observed in dauer (Figure 7) may support this metabolic transition. This could be a strategy adopted to increase the contact area between mitochondria and lipids to accommodate efficient energy supply for the rapid and powerful response in dauer.^[^ ^23^, ^51^ ^]^ This result resembles previous findings of increased lipid droplet abundance and increased mitochondria‐lipid contacts in human skeletal muscles after intense exercise and in neonatal mouse muscles.^[^ ^21^, ^52^ ^]^ While the underlying energy production mechanisms differ as glyoxylate cycle is not present in mammals, these structures appear to be optimized for efficient energy supply suited to their respective metabolic needs.

## 4 Experimental Section

### Electron Microscopy Images

For normal reproductive stages, the study used electron microscopy (EM) images published by Witvliet et al.^[^ ^7^ ^]^ (L1: Dataset 2, L2: Dataset 5, L3: Dataset 6, Adult: Dataset 8). The raw images were acquired from Brain Observatory Storage Service and Database (BossDB) using *intern* library.^[^ ^53^, ^54^ ^]^ For dauer stage, the same EM dataset from Yim et al.^[^ ^30^ ^]^ was used. Please refer to Yim et al.^[^ ^30^ ^]^ for details.

### Volumetric Cell Segmentation

For normal reproductive stages, the study used volumetrically segmented cell reconstructions published by Witvliet et al.^[^ ^7^ ^]^ (L1: Dataset 2, L2: Dataset 5, L3: Dataset 6, Adult: Dataset 8). The segmentation images were acquired from BossDB using *intern* library.^[^ ^53^, ^54^ ^]^ For body wall muscle segmentation, an annotator painted body wall muscles in every 8th section. Then, the intermediate sections were filled by the painted result of the closest section.

Volumetric cell segmentation for dauer images were manually segmented by painting individual sections using VAST.^[^ ^55^ ^]^ Unlike normal reproductive stages, body wall muscles were painted in every section like other cells in the volume.

### Synapses and Connectivity Graph

For normal reproductive stages, the study used synapses and connectivity information published by Witvliet et al.,^[^ ^7^ ^]^ publicly available at BossDB.^[^ ^53^ ^]^ For dauer stage, synapses and connectivity information generated in Yim et al.^[^ ^30^ ^]^ were used.

### Cell Type Classification

Cell types based on neuronal function (sensory, inter‐, motor neurons) were defined according to classification defined in WormAtlas.^[^ ^34^ ^]^ When there are multiple types per neuron, the study defined it as its primary role. The cell type classifications that were used are consistent with what was used in Yim et al.^[^ ^30^ ^]^

Cell types based on the neurotransmitter types (acetylcholine, glutamate, dopamine, GABA, octopamine) were defined according to classification defined in Hobert et al.^[^ ^56^ ^]^ Only primary neurotransmitters were used for classification.

### Ground Truth Annotation for Mitochondria Detection

For normal reproductive stages and the dauer stage EM image stacks, 10 to 20 sections were picked from each stage. All mitochondria in each section were manually labeled using VAST^[^ ^55^ ^]^ and saved as binary images.

### Mitochondria Reconstruction

In every EM image section, mitochondria were detected using deep learning. The network used for the mitochondria detection was adopted from 2D symmetric U‐Net architecture.^[^ ^57^ ^]^ The architecture was composed of five layers with a number of feature maps 16, 32, 64, 128, 256 from the topmost layer to the bottom‐most layer, respectively. For each step in downsampling and upsampling layers, the architecture consisted of three 3 × 3 non‐strided same convolutions. In the downsampling layers, max pooling was used to downsample by a factor of 2 in each layer. In the upsampling layers, a transposed convolution layer with nearest neighbor interpolation followed by 2 × 2 non‐strided convolution was used to upsample by a factor of 2 in each layer. Skip connection was included in every layer which concatenates feature maps at the same level of the left‐hand side to the output of the transposed convolution layer. Instance normalization^[^ ^58^ ^]^ and rectified linear unit (ReLU) was added after each 3 × 3 convolution. At the end of the symmetric network, 3 × 3 non‐strided same convolution and sigmoid function was applied to produce the same‐sized output image, where each pixel value represents probability of each pixel belonging to mitochondria.

The network was trained using 576 × 576 pixel images at an 8 nm resolution. For datasets that did not provide 8 nm resolution, images were downsampled to the closest available resolution so the input to the network would span a comparable field of view.

Separate mitochondria detection models were trained for different datasets. Initial model was trained on ground truth data generated exclusively from the dauer dataset. This initial model has been used as a pretrained model for training the model for L1 stage, and subsequent fine‐tuning was applied to models for the following datasets. The model for adult stage dataset utilized a pretrained model that have been trained on all other datasets.

From the probability map, the image was thresholded with a pixel threshold of 128 / 255 (50%) and generated binarized prediction images of mitochondria. To reconstruct 3D mitochondria, the study applied connected components with connectivity of 26. The errors in resulting reconstructions were corrected manually. All the mitochondria were skeletonized using Kimimaro.^[^ ^59^, ^60^ ^]^

The study conducted two approaches to evaluate the quality of the mitochondria reconstructions, a pixel‐based evaluation and an object‐based evaluation. For the pixel‐based evaluation, each thresholded pixel of prediction image was compared to the ground truth to calculate precision and recall in a test data, distinct from the training data. For the object‐based evaluation, all the mitochondria in the test cutout were manually segmented and labeled as the ground truth. The study then checked whether each segmented mitochondrion was detected in the reconstructed output to compute precision and recall. The object‐based evaluation was performed only on the dauer dataset, where the pixel‐based results were the lowest.

### Computation of Synaptic Properties

Out‐ and in‐degrees were calculated by counting the total number of postsynaptic and presynaptic partners respectively. For example, if there's 3 postsynaptic partners for an active zone, 3 will be added to compute out‐degree. Synapse size was approximated by measuring the size of the active zone. The study counted the number of voxels, then multiply by the voxel resolution to get the physical size of an active zone. In this paper, the terms “active zone size” and “synapse size” were used interchangeably. Fan‐out is a measure that is computed for each active zone, indicating the number of postsynaptic partners per active zone. Cells with either 0 out‐ or in‐degree were neglected from the corresponding analyses.

### Computation of Mitochondria Properties

Number of mitochondria was the total number of mitochondria in a cell. Mitochondria volume indicated the size of each mitochondrion. The volume was calculated by multiplying the voxel count by the voxel resolution. For analyses done per cell, mitochondria volume means total sum of volumes of all mitochondria included in a cell (Figures 5, 6, 7). Mitochondria volume fraction is a proxy for mitochondria density of each cell. The volume fraction was computed by dividing the total mitochondria volume in a cell by the volume of a cell. Mitochondria surface area per volume (SAV) was calculated by dividing the total sum of surface area of all mitochondria by the total sum of volumes of all mitochondria. To compute the amount of mitochondria branching in muscle cells, the study pseudo‐randomly sampled five sections among sections where the cell lies and counted the number of mitochondrial cross‐sections in each section. Mitochondrial complexity index (MCI)^[^ ^42^ ^]^ was computed as

(1) $$MCI=\frac{SA^{3}}{16\pi^{2}V^{2}}$$

where *SA* is the surface area and *V* is the volume of mitochondria. Cells without any mitochondria have been neglected from the corresponding analyses.

### Computation of Distance Between Synapses and Mitochondria

Mitochondria positions were determined by the centroids of segmented mitochondria and the positions of presynaptic sites were determined by the centroids of segmented active zones. For postsynaptic sites, the positions were assigned to the active zone positions for corresponding presynaptic partners. Depending on the analysis, the study either measured distance to nearest mitochondria from pre‐ ($d_{mito}^{pre}$) and postsynaptic ($d_{mito}^{post}$) sites, or distance from each mitochondria to nearest pre‐ ($d_{syn}^{pre}$) and postsynaptic ($d_{syn}^{post}$) sites by calculating the euclidean distance between two positions.

For randomizations used in Figure 2D, random mitochondria was assigned within the same neuron instead of the nearest mitochondria for each active zone and measured the distance. 1000 random configurations have been generated and mean and 95% confidence intervals are shown in Figure 2D. In Figure 2E, distribution of distances from one randomly selected configuration is shown as representative.

To distinguish nearby and far mitochondria from synapses as in Figure 2F,G, the study set the threshold to be the distance where the cumulative synapse distribution starts to saturate (<5% difference). The resulting threshold distances were 1.3 µm (L1), 1.3 µm (L2), 1.1 µm (L3), 1.1 µm (adult), and 1.1 µm (dauer).

### Microdroplet Swimming Assay

A microdroplet swimming assay was performed to simultaneously measure the frequency of reorientation in many nematodes. Young adult animals were grown on standard nematode growth medium plates containing *E*. *coli* OP50. Nematodes in the food lawn were washed in M9 solution to remove *E*. *coli* attached to their bodies, and their behavior was measured by placing them in 1.5 µL of M9. Swimming and reorientation behavior videos were recorded at 10 Hz. Custom algorithm was used to measure turning rates in micro‐droplets.^[^ ^61^ ^]^ The experiments were only conducted in the adult stage since turns are difficult to detect in other developmental stages as the worms are small. Besides, the worms tend to stay motionless in the dauer stage.

### Principal Component Analysis of Mitochondrial Features

To investigate the overview of mitochondria features in neurons, eight structural features of mitochondria were used: Total mitochondria volume, mean mitochondria volume, total mitochondria surface area, mean mitochondria surface area, total mitochondria length, mean mitochondria length, mitochondria volume fraction, and mean mitochondrial complexity index. The features were standardized and projected onto the first two principal components.

### UMAP Analysis of Mitochondrial Features

To investigate the overview of mitochondria features in body wall muscles, five structural features of mitochondria were used: Total mitochondria volume, total mitochondria surface area, mitochondria volume fraction, mitochondria surface area per volume, and mitochondrial complexity index. The features were standardized and projected onto the first two components of UMAP embeddings.^[^ ^45^ ^]^

### Sample Preparation and Fluorescence Imaging

Confocal microscopy (ZEISS LSM700; Carl Zeiss) was used to observe mitochondrial transgene expression in the body wall muscle of *C. elegans*. For microscopy and imaging, transgenic animals were paralyzed with 3 mM levamisole and mounted on 3% agar pads. All transgenic animals were observed and imaged at the stage as described: L1, L2, L3, L4, Day 0 adult, Day 5 adult, dauer, Day 0 postdauer adult, Day 5 postdauer adult. All stage worms were collected after *C. elegans* synchronization (L1) with cultivation during proper developmental time except dauer and postdauer adult.

### Neurons and Mitochondria Renderings

All the renderings of neurons and mitochondria were created in Python using MeshParty (github.com/CAVEconnectome/MeshParty). Screenshots of Neuroglancer (github.com/google/neuroglancer) were used for some figures (Figure S6, Supporting Information).

### Statistical Analysis

To find the significance in linear correlation, Pearson correlation was used (Figure 2; Figure S3, Supporting Information). For comparing two independent distributions, Wilcoxon rank‐sum test was used as most of the distributions did not follow the normal distribution (Figures 2, 3, 4, 5, 6, 7; Figures S3, S8, and S9, Supporting Information). Significance tests with *p*‐values < 10^−20^ were marked as *P* ≈ 0.

## Conflict of Interest

The authors declare no conflict of interest.

## Author Contributions

J.A.B. and M.C. contributed equally to this work. K.C.N and D.H.H. acquired dauer EM image stack. G.K., H.Y., and D.T.C. refined the cell segmentation of dauer dataset. J.A.B. trained the mitochondria detection models and reconstructed mitochondria using the ground truth annotated by G.K. J.A.B. performed computational analysis. M.C. designed and implemented the microdroplet swimming assay and performed behavior experiments with help from D.T.C. S.A., M.C., and D.T.C. acquired fluorescence images of mitochondria. J.A.B. wrote the paper with contributions from M.C., D.H.H., J.S.K., and J.L. J.A.B., J.S.K., D.H.H., and J.L. managed the multi‐institution collaboration.

## Supporting information



Supporting Information

## Data Availability

All data needed to evaluate the conclusions in the paper are present in the paper and/or the Supplementary Materials. Mitochondria reconstructions along with EM images, cell segmentation images, and connectivity data for normal reproductive stages are publicly available at https://bossdb.org/project/witvliet2020 (https://doi.org/10.60533/BOSS‐2020‐FQI7). The data for the dauer stage are publicly available at https://bossdb.org/project/yim_choe_bae2023 (https://doi.org/10.60533/BOSS‐2023‐RTPH). All the data can be downloaded and viewed on BossDB. Mitochondria reconstruction and analysis codes are available at https://github.com/jabae/Cmito.
